# Rapid screening for antibiotic resistance elements on the RNA transcript, protein and enzymatic activity level

**DOI:** 10.1186/s12941-016-0167-8

**Published:** 2016-09-23

**Authors:** Alexander Rohde, Jens Andre Hammerl, Sascha Al Dahouk

**Affiliations:** 1 Department of Biological Safety, Federal Institute for Risk Assessment, Diedersdorfer Weg 1, 12277 Berlin, Germany; 2 Department of Biology, Chemistry and Pharmacy, Free University Berlin, Takustr. 3, 14195 Berlin, Germany

**Keywords:** Antibiotic resistance, Fluorescence in situ hybridization, β-Lactamases, Plasmids

## Abstract

**Background:**

The emerging threat posed by antibiotic resistance has affected public health systems all over the world. Surveillance of resistant bacteria in clinical settings and identifying them in mixed cultures is of paramount importance and can contribute to the control of their spreading. Culture-independent monitoring approaches are highly desirable, since they yield results much faster than traditional susceptibility testing. However, many rapid molecular methods like PCR only detect the sole presence of a potential resistance gene, do not provide information regarding efficient transcription, expression and functionality and, in addition, cannot assign resistance genes to species level in mixed cultures.

**Methods:**

By using plasmid-encoded TEM β-lactamase mediated ampicillin resistances as a proof of principle system, we (1) developed a fluorescence in situ hybridization-test (FISH) capable to detect the respective mRNAs, (2) implemented an immunofluorescence test to identify the corresponding proteins and (3) compared these two microscopic tests with an established colorimetric nitrocefin assay to assess the enzymatic activity.

**Results:**

All three methods proved to be suitable for the testing of antibiotic resistance, but only FISH and immunofluorescence were able to differentiate between susceptible and resistant bacteria on the single cell level and can be combined with simultaneous species identification.

**Conclusions:**

Fluorescence in situ hybridization and immunofluorescence tests are promising techniques in susceptibility testing since they bridge the gap between the slow, but accurate and sound cultural methods and molecular detection methods like PCR with much less functional relevance.

**Electronic supplementary material:**

The online version of this article (doi:10.1186/s12941-016-0167-8) contains supplementary material, which is available to authorized users.

## Background

The emergence of antibiotic resistance is threatening the public health in industrialized as well as developing countries, resulting in therapy failure and increased health-care expenditures [1, 2]. Monitoring the occurrence of antibiotic resistance is one important component to control its spreading and, consequently, numerous surveillance programs have been implemented [3–6]. The susceptibility of bacterial isolates towards certain antimicrobials is mainly assessed by slow cultural methods, which require the availability of pure isolates. However, in clinical settings rapid susceptibility testing is extremely crucial to initiate appropriate therapeutic measures because any delay might increase morbidity, mortality and long-term sequelae; therefore, alternative testing methods are gaining importance in hospital care [7]. Screening ubiquitous biofilms in hospitals consisting of multiple different bacterial species or blood samples containing mixed cultures for resistance genes can be carried out by PCR-testing; however, detected resistance genes can neither be attributed to a specific species, nor can it prove a functional resistance. Therefore, culture-independent assays on the single cell level, preferably coupled with the simultaneous species identification, are highly desirable, not only for academic purposes, but also in clinical or environmental microbiology. β-lactamases are one of the most frequently encountered mediators of antimicrobial resistance. Among them, TEM β-lactamases encoded on plasmids represent especially interesting targets because mobile elements like plasmids can easily spread antibiotic resistance. To detect the presence of these resistance elements, three culture-independent methods appear to be capable of fulfilling this task. Firstly, the detection of TEM β-lactamase mRNA transcripts can be performed by a modified version of fluorescence in situ hybridization (FISH), which was initially developed for eukaryotes and has recently also been successfully used for prokaryotes [8–10]. Previous attempts using FISH were mainly restricted to detect single mutations in the highly abundant rRNAs conferring resistance to antibiotics targeting ribosomes, e.g. macrolides [11, 12]; however, by applying dozens of probes simultaneously (instead of only one) mRNAs with much lower concentrations can be detected as well. In parallel with mRNA detection, FISH can be used to simultaneously identify the bacterial species based on their ribosomal RNA [7, 11]. Secondly, immunofluorescence stainings of the proteins with specific antibodies represent a further alternative and can also be used for concurrent species identification [13]. Thirdly, chromogenic substrates like nitrocefin offer a way to test the enzymatic activity of TEM β-lactamases [14, 15]. Apart from these methods, reverse transcription PCR assays (RT-PCR) are an additional possibility enabling the detection of efficient transcription [16–18]. However, in contrast to FISH and immunofluorescence stainings, RT-PCR cannot detect resistance elements on the single cell level. In this work, we established a FISH assay to detect TEM mRNAs encoded on different kind of plasmids and, additionally, an immunofluorescent staining to detect the corresponding proteins and compared these methods with the traditional nitrocefin assay to screen for functional β-lactamases.

## Methods

### Strains and cultivation

*Escherichia coli* strain DH5α carrying either the high copy number plasmids pLitmus38 (AMP^r^) and pUC18 (AMP^r^) or the medium copy number vector pBR328 (AMP^r^, TET^r^, CHL^r^) with a low plasmid stability were used as well as the *E. coli* reference strain ATCC 35218, a reference strain producing TEM-1 β-lactamases [19–21]. On the plasmids pLitmus38, pUC18 and pBR328 a TEM-1 gene confers resistance towards ampicillin. Susceptibility of these bacterial strains was tested by Etest® (bioMerieux, France) according to manufacturer specifications and all AMP^r^*E. coli* strains possessed MICs >256 µg/ml. As negative controls, DH5α (MIC <1.5 µg/ml) and GeneHogs (MIC <2 µg/ml; Thermo Fisher Scientific, USA) without plasmids were used as susceptible *E. coli* strains as well as *Y. pseudotuberculosis* ATCC 29833 (MIC <0.125 µg/ml). Two clinical *Klebsiella pneumoniae* isolates, K2 with an intermediate resistance (MIC <24 µg/ml) and the highly resistant strain My6107 (MIC >256 µg/ml), and *Yersinia enterocolitica* DSM 13030 (MIC >256 µg/ml) were used as ampicillin-resistant strains expressing non-TEM β-lactamases. *E. coli* and *K. pneumoniae* were grown in LB medium at 37 °C, *Y. enterocolitica* and *Y. pseudotuberculosis* at 28 °C. To exhibit antibiotic stress, bacterial cultures were grown in LB medium containing ampicillin in a concentration of 100 µg/ml.

In addition, 25 *E. coli* isolates from different environmental samples and with different TEM-variants (Additional file 1: Figure S1) were used to verify the inclusivity and sensitivity of the established FISH and immunofluorescence assays. To test whether the newly developed assays are applicable for mixed bacterial cultures, samples containing different species (*Salmonella enterica*, thermophilic *Campylobacter* spp., *Listeria* spp., *Y. enterocolitica*, *E. coli* O157) were prepared as described earlier [22].

### Fish

Bacterial cultures were fixed by adding three volumes of 4 % (vol/vol) PBS/formaldehyde mixture (Carl Roth, Germany). Samples were incubated for 2 h at 4 °C and then washed three times by centrifugation and resuspension in PBS. Cells were resuspended in a 50 % Ethanol/PBS (vol/vol) mixture and either used directly or stored at −20 °C. 10 µl of each sample were pipetted on glass slides (miacom® diagnostics, Germany), dried shortly on a 52 °C hot plate (miacom® diagnostics) and dehydrated in 50, 80 and 96 % ethanol for 3 min each. The slides were coated with 10 μl hybridization buffer (1 M NaCl, 20 mM Tris–HCl (pH 7.2), 0.01 % SDS, 15 % formamide) containing FISH probes in an accumulated concentration of 800 nM (or approximately 20 nM for each FISH probe). FISH probes hybridized in a light-protected humidity chamber at 30 °C for at least 1.5 h. Slides were rinsed in distilled cold water for a few seconds, followed by washing for 10 min (310 mM NaCl, 20 mM Tris–HCl (pH 7.2), 0.01 % SDS) at 30 °C. Slides were again rinsed twice in distilled water, immediately air-dried and embedded in Roti®-Mount FluorCare DAPI (Carl Roth). FISH probes were designed with the Stellaris Probe Designer and synthesized and labelled with CalFluor Red 610 by Biosearch Technologies (Petaluma, USA). The *bla*-gene of pBR328 (GenBank accession #: L08858.1) was used to construct the FISH probes as listed in Table 1. A conventional FISH probe developed by Bohnert et al. targeting the ribosomes of Enterobacteriaceae (Enterobac and the unlabeled competitor Enterobac-Komp) was used as a positive hybridization control (Table 1; [23]). FISH probe lyophilisates were diluted in distilled water and stored at −20 °C until usage. Each bacterial strain was tested in three independent hybridization experiments.

**Table 1:** Oligonucleotides used to detect TEM mRNA and Enterobacteriaceae (as a positive hybridization control)

Probe name	Sequence (5′→3′)	Target	Detection purpose
Enterobac-Alexa488	TCGTGTTTGCACAGTGCTGTGTTT	23S rRNA	Enterobacteriaceae (adapted with minor modifications from Bohnert et al. [23])
Enterobac-Komp	TCGTGTTTGCAGAGTGCTGTGTTT	23S rRNA	Competitor for Enterobacteriaceae detection (adapted with minor modifications from Bohnert et al. [23])
Bla-TEM-CalFluor610	GGAAATGTTGAATACTCAT AAAAGGGAATAAGGGCGAC CAGGAAGGCAAAATGCCGC GCGTTTCTGGGTGAGCAAA CAGCATCTTTTACTTTCAC CTCGTGCACCCAACTGATC GATCCAGTTCGATGTAACC CAAGGATCTTACCGCTGTT GTTCTTCGGGGCGAAAACT AAGTGCTCATCATTGGAAA CGCCACATAGCAGAACTTT CGTCAACACGGGATAATAC GACCGAGTTGCTCTTGCCC TCTGAGAATAGTGTATGCG GTGAGTACTCAACCAAGTC TAAGATGCTTTTCTGTGAC CTCTTACTGTCATGCCATC TTATGGCAGCACTGCATAA CCGCAGTGTTATCACTCAT TCGTTGTCAGAAGTAAGTT TTAGCTCCTTCGGTCCTCC CCATGTTGTGCAAAAAAGC CAAGGCGAGTTACATGATC TCAGCTCCGGTTCCCAACG CGTCGTTTGGTATGGCTTC CAGGCATCGTGGTGTCACG GCAACGTTGTTGCCATTGC GTTCGCCAGTTAATAGTTT GCCGGGAAGCTAGAGTAAG CCATCCAGTCTATTAATTG GTCCTGCAACTTTATCCGC GAAGGGCCGAGCGCAGAAG CAGCAATAAACCAGCCAGC GCTCACCGGCTCCAGATTT CAATGATACCGCGAGACCC TACCATCTGGCCCCAGTGC TAACTACGATACGGGAGGG CCTGACTCCCCGTCGTGTA TATTTCGTTCATCCATAGT CACCTATCTCAGCGATCTG ACCAATGCTTAATCAGTGA	mRNA	TEM β-lactamase mRNA

### Immunofluorescence

Bacterial cultures were prepared as described for FISH. After drying on a glass slide, the bacteria were permeabilized with lysozyme (Carl Roth, 10 mg/ml) for 7 min and afterwards rinsed shortly with water. Samples were then blocked with blocking buffer [2 % of bovine serum albumin in PBS (Sigma-Aldrich, USA)] for 1 h at room temperature. Subsequently, the primary antibody [anti-(TEM) β-lactamase ab12251 (mouse); abcam, United Kingdom], diluted 1:200 in blocking buffer, was added and incubated either overnight at 6 °C (for sequential stainings) or 1 h at room temperature. Slides were rinsed shortly with water and washed three times with PBS and, finally, with blocking buffer for 3 min each. Slides were then incubated with the secondary antibody (goat anti-mouse IgG-Alexa Fluor® 488 ab150117, abcam), diluted 1:300 in blocking buffer, for 1 h at room temperature. Slides were again rinsed shortly with water and washed three times with PBS for 3 min each, once more rinsed with water, air-dried and embedded in Roti®-Mount FluorCare DAPI. Each bacterial strain was stained in three independent immunofluorescence assays.

### Fluorescence microscopy

Microscopy was carried out with an AxioScope fluorescence microscope using a 100× N-achroplan Ph3 M27 oil objective (Zeiss, Germany). Images were acquired by the AxioCam MRm and further processed for overlay of different fluorophore channels by using the imaging software ZEN 2012 (Zeiss).

### Nitrocefin assay

Nitrocefin (Merck Millipore, Germany) was dissolved in DMSO (PanReac Applichem, Germany) in a concentration of 5 mg/ml. 50 µl of this stock solution was added to 950 µl PBS. 50 µl of this nitrocefin working solution was added to 150 µl of a bacterial culture. Alternatively, a colony was picked from an agar plate and suspended in 50 µl nitrocefin working solution on a glass slide. A colour change from yellow to red was considered as proof for functional β-lactamases. Each bacterial strain was tested three times.

## Results and discussion

**Fig. 1:**
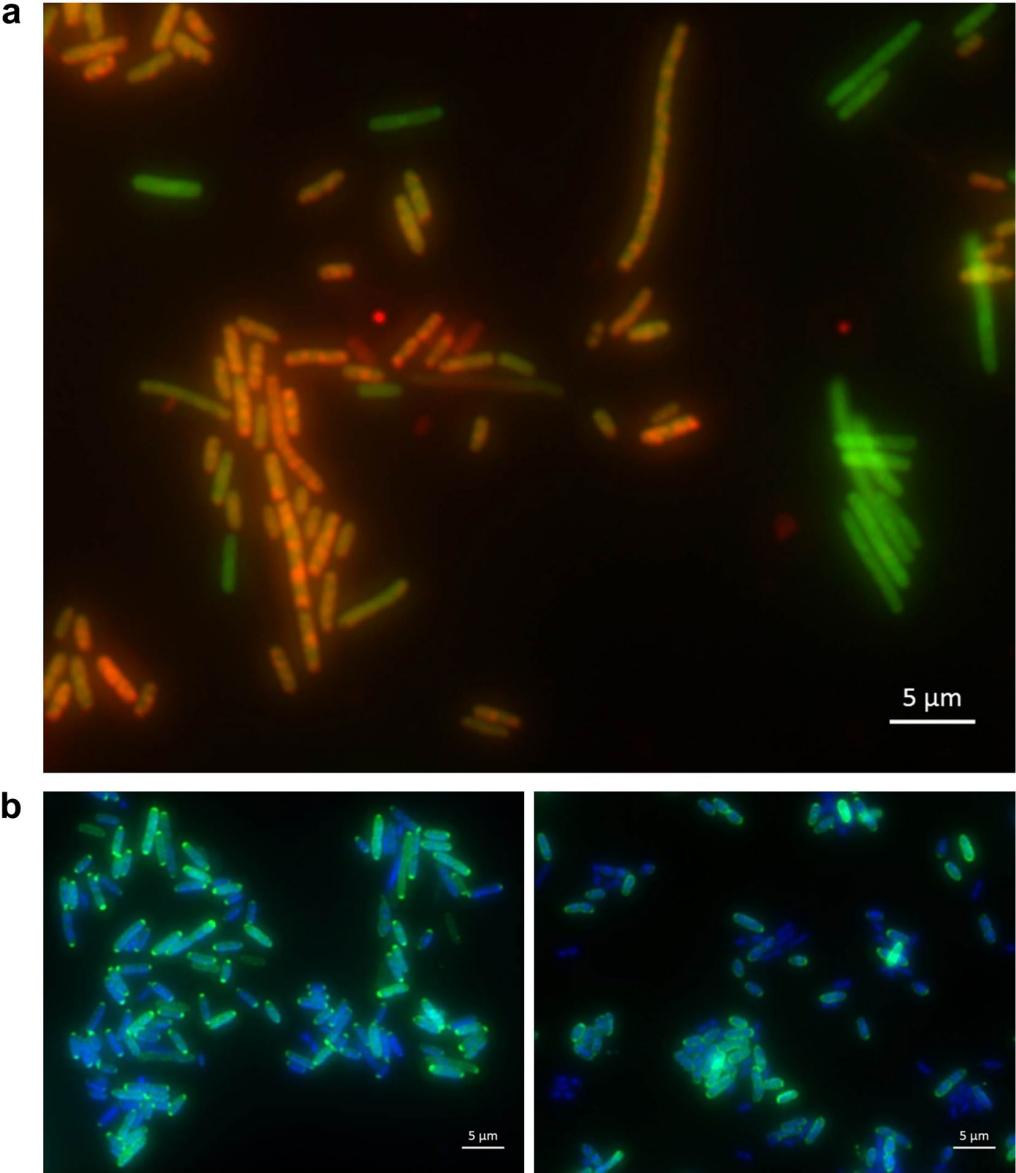
Detection of TEM β-lactamase mRNAs and the respective protein in *E. coli*. **a** FISH staining of TEM β-lactamase mRNA in *E. coli* harboring pLitmus38 (*red*) and the ribosomal RNAs by Enterobac (*green*). **b** Antibody (*green*) and DNA/DAPI (*blue*) staining of the TEM β-lactamases in *E. coli* harboring pLitmus38 (*left*) or pUC18 plasmids (*right*)

The FISH assay proved to be sensitive enough to detect signals in all TEM β-lactamase producing strains. FISH signals were highly specific and showed no hybridization with susceptible *E. coli* strains without plasmids conferring resistance or with species which possess other types of β-lactamases (Additional file 2: Figure S2). Interestingly, the transcription pattern varied among individual cells of a pure culture (Fig. 1a) and depended on the tested plasmids as well as on the growth phase: *E. coli* strains harbouring pLitmus38 and pUC18 showed significantly stronger signals than *E. coli* ATCC 35218 or strains with pBR328. In addition, stationary cultures exhibited stronger FISH signals than exponentially growing cultures, which might be a result of a slower metabolism or prolonged mRNA half-lives. The FISH assay could be easily combined with conventional rRNA-FISH for bacterial identification, as exemplified by the simultaneous use of the FISH probe Enterobac. Notably, not all cells which were stained via conventional ribosome FISH staining showed detectable transcription rates of the TEM β-lactamase (Fig. 1a), which can be explained with a natural variation in the transcription rates on the single cell level, a phenomenon which has been observed for other mRNAs before [9, 10]. In contrast to the ribosome staining by the conventional FISH probe Enterobac, the mRNA signal was not evenly distributed throughout the bacterial cell. Instead, several distinct foci can be observed (Fig. 1a), which is in accordance with previously published reports about mRNA distribution in prokaryotes [9, 10].

Immunofluorescence staining of the TEM β-lactamase protein showed a more even signal distribution among the bacterial cells than the RNA-signals determined by mRNA FISH (compare Fig. 1a, b). Stationary cultures also showed stronger signals compared to exponentially growing bacteria. As expected for a protein which is secreted in the periplasm, ring-shaped halo-like structures around the cells were observed, especially for pUC18 *E. coli* strains (Fig. 1b, right), whereas strains harbouring pLitmus38 and pBR328 as well as *E. coli* ATCC 35218 showed protein accumulations mainly in the cell poles (Fig. 1b, left). In accordance with FISH, antibody staining produced stronger signals for pLitmus38 and pUC18 than for pBR328 and *E. coli* ATCC 35218. Bacteria without a TEM gene were not stained by immunofluorescence (Additional file 3: Table S1).

**Fig. 2:**
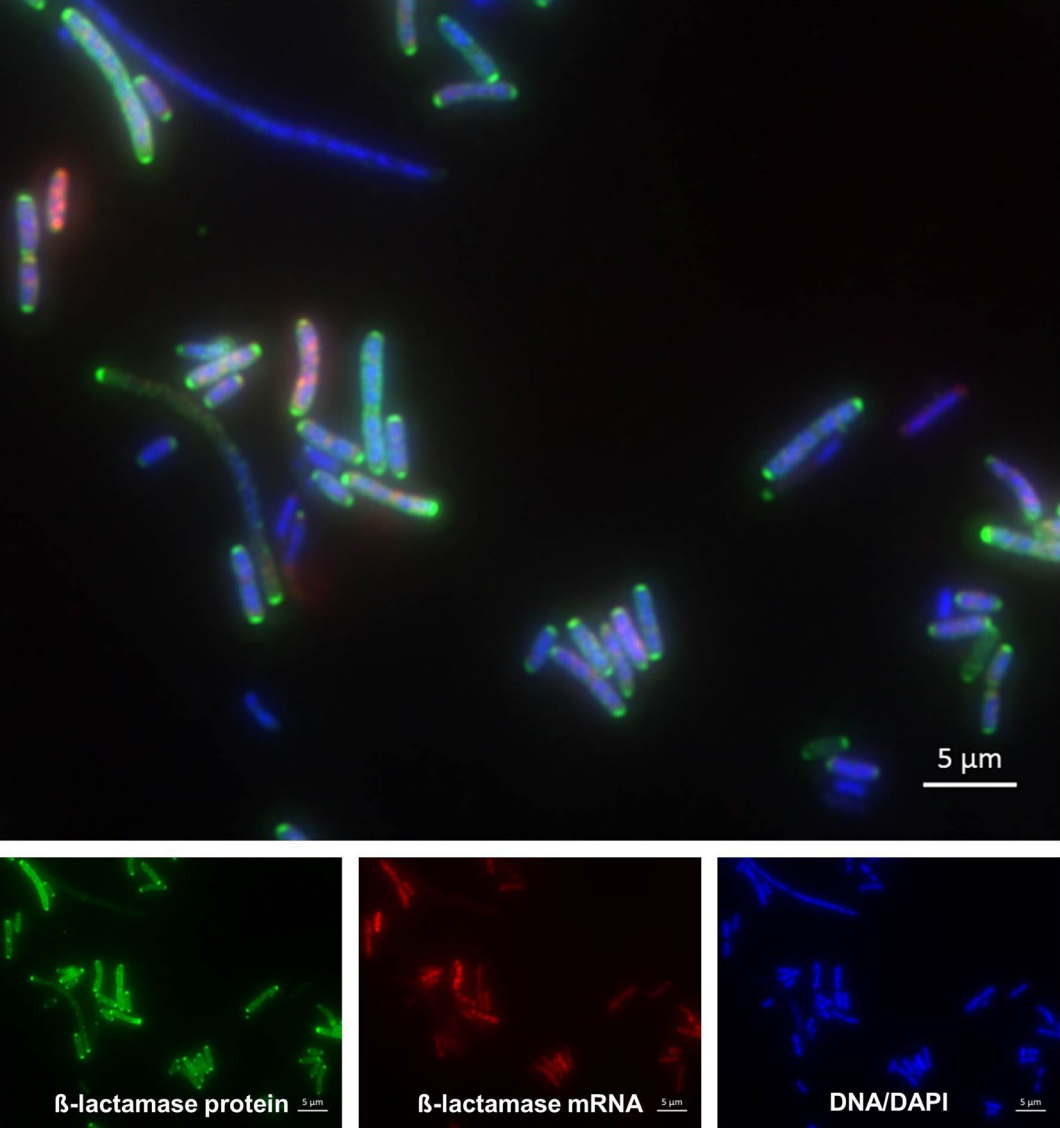
Simultaneous staining of TEM mRNA and TEM protein in *E. coli* harboring pLitmus38. The upper image shows the merge of the mRNA signal (*red*), the protein level (*green*) and the DNA/DAPI staining (*blue*). The three images below show each fluorescence channel separately

To elucidate the correlation between transcription and protein level, a sequential FISH and immunofluorescence staining was performed (Fig. 2). However, a clear association between both signals could not be inferred. Some cells showed a pronounced immunofluorescence signal, thus detectable amounts of protein, but a negative FISH signal (thus no detectable mRNAs) or vice versa.

To verify that both assays can be applied to environmental samples, 25 *E. coli* strains with different TEM-variants (TEM-1, TEM-30, TEM-52) were tested. All 25 isolates were detected by immunofluorescence and showed protein accumulations mainly in the cell poles. However, four strains exhibited a rather weak antibody staining. Likewise, the FISH signals of two strains were too weak to be reliably detected (Additional file 1: Figure S1). However, a combination of both methods enabled clear results. This combined testing might be useful to avoid false-negative results and is especially advisable if samples with strong background fluorescence are examined like food matrices or filtrates [22, 24].

**Fig. 3:**
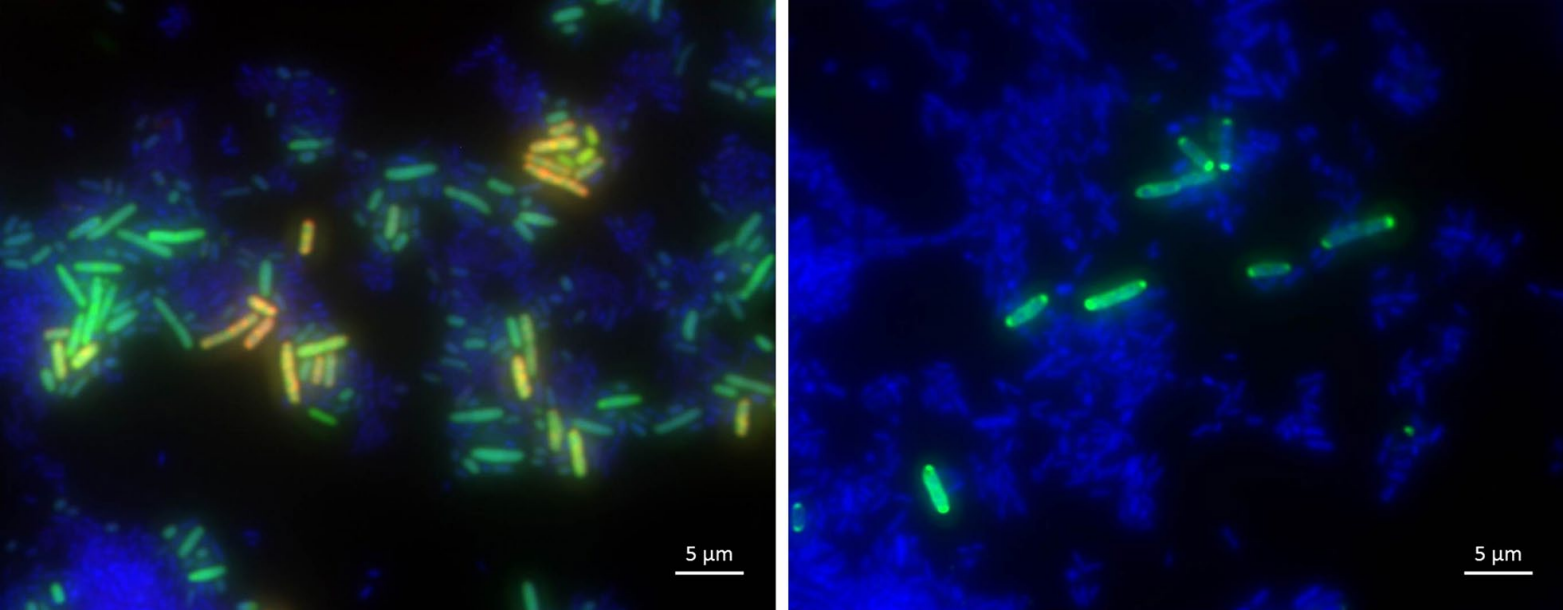
Detection of resistance in mixed microbial samples. *Left* FISH staining of TEM β-lactamase mRNA in resistant *E. coli* (*red*), ribosomal RNA staining of all Enterobacteriaceae (*green*) and DNA/DAPI staining of all bacteria in the sample. *Right* Antibody staining of TEM β-lactamases expressing *E. coli* (*green*) and DNA/DAPI staining of all bacteria in the sample

To demonstrate that the detection of antibiotic resistance in samples with many different species is possible, mixed microbial cultures were prepared containing a resistant *E. coli* strain as well as *Listeria* spp., *Campylobacter* spp. and Enterobacteriaceae like *Y. enterocolitica*, *S. enterica* and susceptible *E. coli* (all without TEM-elements). Both methods, FISH and immunofluorescence, reliably identified resistant bacteria within this mixture (Fig. 3).

The nitrocefin assay is an established general assay to identify the presence of most types of β-lactamase producing strains. All *E. coli* strains with the high copy number plasmids pUC18 and pLitmus38 as well as *K. pneumoniae* My6107 were rapidly tested positive for β-lactamase in a few minutes, both by colony smear as well as in liquid cultures, whereas all susceptible strains produced negative results (Additional file 4: Figure S3). Interestingly, the colour change for *Y. enterocolitica*, *K. pneumoniae* K2, *E. coli* ATCC 35218 and strains harbouring the plasmid pBR328 with a low stability and a relatively weak β-lactamase production [20] was less pronounced, especially in liquid cultures without selection pressure, and took significantly longer than for the resistant *E. coli* strains with high copy plasmids, which showed a much more rapid substrate turnover (Additional file 4: Figure S3). We also tried to use nitrocefin to visualize β-lactamase activity on the single cell level. However, nitrocefin was not retained within the periplasm and was, thus, unable to distinguish resistant from susceptible cells using microscopy. It has to be noted that also fluorogenic β-lactamase substrates like CCF2-AM are available, but these substrates are much more expensive and their application is mainly limited to eukaryotic reporter gene assays.

In summary, all three methods are able to detect antibiotic resistance, in particular in case of constitutive moderate transcription and expression rates, and produce congruent results with regard to signal strength. However, there are intrinsic strength and weaknesses of each technique (Table 2). The fast, affordable and easy nitrocefin activity assay can sense the presence of a broad range of β-lactamases and is not confined to TEM-like proteins. In addition, detecting biological activity is probably the most meaningful way to search culture-independently for antibiotic resistance. However, nitrocefin is hardly suitable for single cell microscopy, is not very sensitive in the presence of only low numbers of resistant bacteria and, in contrast to FISH and immunofluorescence, cannot be used for the simultaneous species identification on the single cell level. The β-lactamase antibody staining proved to be a highly convenient and robust system, yielding strong and specific signals. However, it is the slowest of all three methods and depends on the availability of suitable (and rather costly) antibodies. Mutations, which are frequent events in the evolution of antibiotic resistance genes, might easily compromise antibody binding. Finally, multi-probe mRNA FISH assays employing up to 50 probes per target mRNA are able to effectively detect groups of related gene products. Efficient detection of mRNAs has been previously performed by RT-PCR; the additional advantage of FISH, however, lies in the detection of resistance on the single cell level, enabling refined insights in multi-species mixed communities and samples. Recently, we developed an extensive set of free-combinable FISH probes targeting the rRNA of various pathogenic bacteria [22]. The assay presented here is a valuable enhancement of these tests. Convenient mRNA FISH assays are not only conceivable to screen for antibiotic resistance elements but are also promising tools to identify the transcription of toxins and other virulence factors. In contrast to the detection by antibodies, mutations are unlikely to affect the detection via FISH, since the use of 50 probes or more tolerates hybridization failure of a few probes. However, monitoring the transcription has the least biological relevance and does neither prove efficient translation nor sufficient biological effectivity.

**Table 2:** Comparison of culture-independent techniques applied for the screening of antibiotic resistance

Detection method	FISH	Immunofluorescence	Nitrocefin assay
Target	mRNA	Protein	Enzymatic activity
Speed (h)	5	7	<1
Costs	Moderate	Moderate/high	Low
Simultaneous species identification	Possible	Possible	Not possible
Specificity	Narrow, type-specific (e.g. TEM)	Narrow, type-specific (e.g. TEM)	Broad
Drawbacks	Transcription is no correlate for efficient expression	Specific antibodies required	Not suitable on the single cell level

## Conclusions

Fluorescence in situ hybridization and immunofluorescence tests represent promising and affordable tools for susceptibility testing on the single cell level. They combine the speed of other rapid and culture-independent methods with the ability of the cultural methods to obtain functional information and, furthermore, have the potential for simultaneous species identification.

## Additional files

10.1186/s12941-016-0167-8 Specificity testing of the mRNA FISH assay.
10.1186/s12941-016-0167-8 Specificity testing of the immunofluorescence assay.
10.1186/s12941-016-0167-8 Examined environmental *E. coli* isolates with TEM-mediated ampicillin resistance.
10.1186/s12941-016-0167-8 Detection of ß-lactamase activity by the nitrocefin assay.

## Abbreviations

FISH: fluorescence in situ hybridization
PCR: polymerase chain reaction
RNA: ribonucleic acid
mRNA: messenger RNA
rRNA: ribosomal RNA
RT-PCR: reverse transcription PCR
AMP^r^: ampicillin resistance
TET^r^: tetracycline resistance
CHL^r^: chloramphenicol resistance
MIC: minimal inhibitory concentration
*Y. pseudotuberculosis*: *Yersinia pseudotuberculosis*
*E. coli*: *Escherichia coli*
*K. pneumoniae*: *Klebsiella pneumoniae*
*Y. enterocolitica*: *Yersinia enterocolitica*
LB: lysogeny broth
PBS: phosphate-buffered saline
DAPI: 4′,6-diamidino-2-phenylindole
DMSO: dimethyl sulfoxide
ATCC: American Type Culture Collection
