# Supplementary material for: Rapid screening for antibiotic resistance elements on the RNA transcript, protein and enzymatic activity level
Source: Ann Clin Microbiol Antimicrob. 2016 Sep 23;15:55. doi: 10.1186/s12941-016-0167-8 (PMC5035493; doi:10.1186/s12941-016-0167-8)
Supplement: Supplementary file 1 — 10.1186/s12941-016-0167-8 Specificity testing of the mRNA FISH assay. [file 12941_2016_167_MOESM1_ESM.docx]

**Supplementary Data I**

Additional File 1: Figure S1: Specificity testing of the mRNA FISH assay


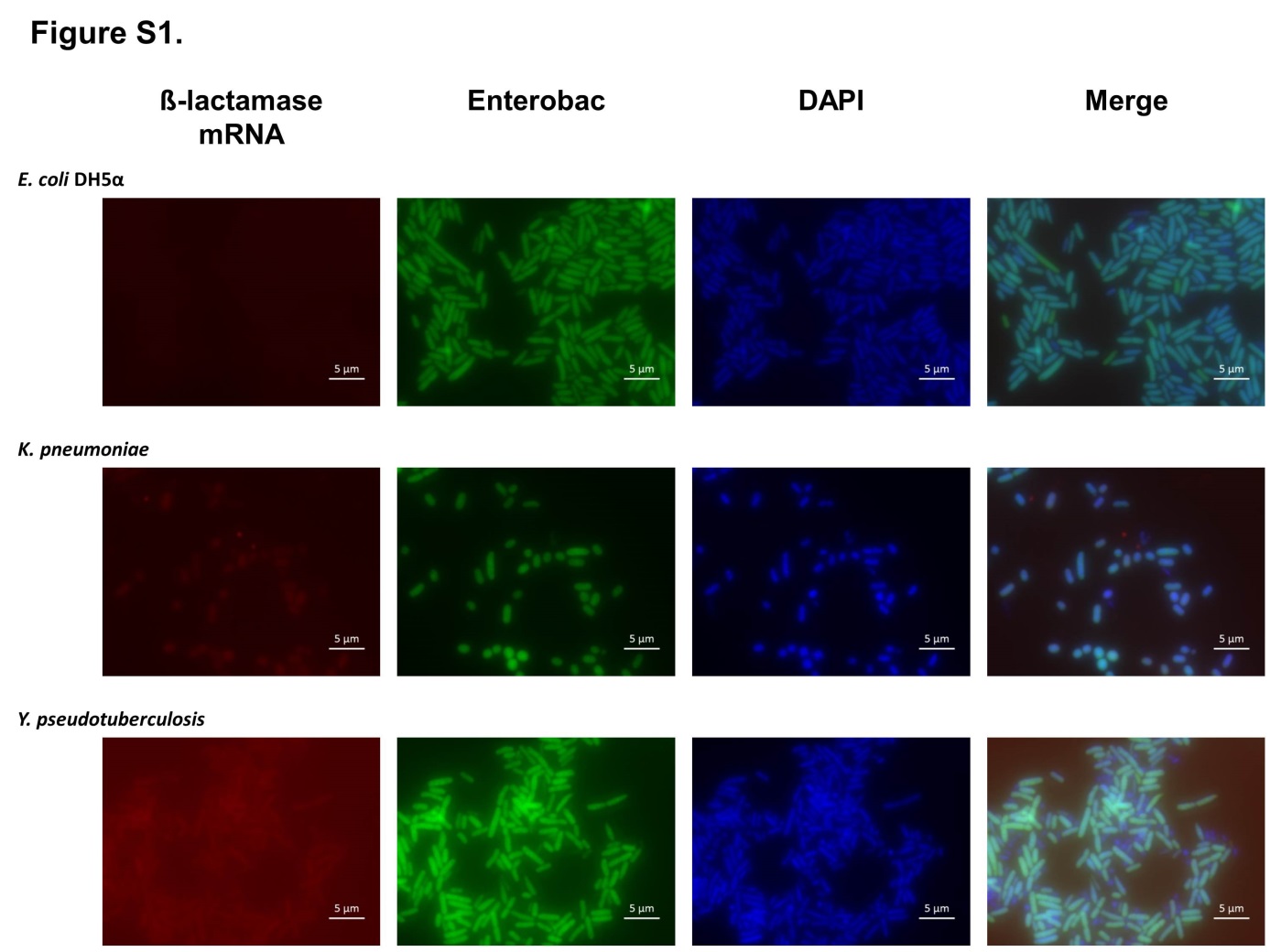


FISH staining of TEM ß-lactamase mRNA (red), FISH staining of the ribosomal RNAs by Enterobac (green) and DAPI (blue) staining. First row: *E. coli* DH5α. Second row: *K. pneumoniae*. Third row: *Y. pseudotuberculosis*.
