# Supplementary material for: Rapid screening for antibiotic resistance elements on the RNA transcript, protein and enzymatic activity level
Source: Ann Clin Microbiol Antimicrob. 2016 Sep 23;15:55. doi: 10.1186/s12941-016-0167-8 (PMC5035493; doi:10.1186/s12941-016-0167-8)
Supplement: Supplementary file 2 — 10.1186/s12941-016-0167-8 Specificity testing of the immunofluorescence assay. [file 12941_2016_167_MOESM2_ESM.docx]

**Supplementary Data II**

Additional File 2: Figure S2: Specificity testing of the immunofluorescence assay


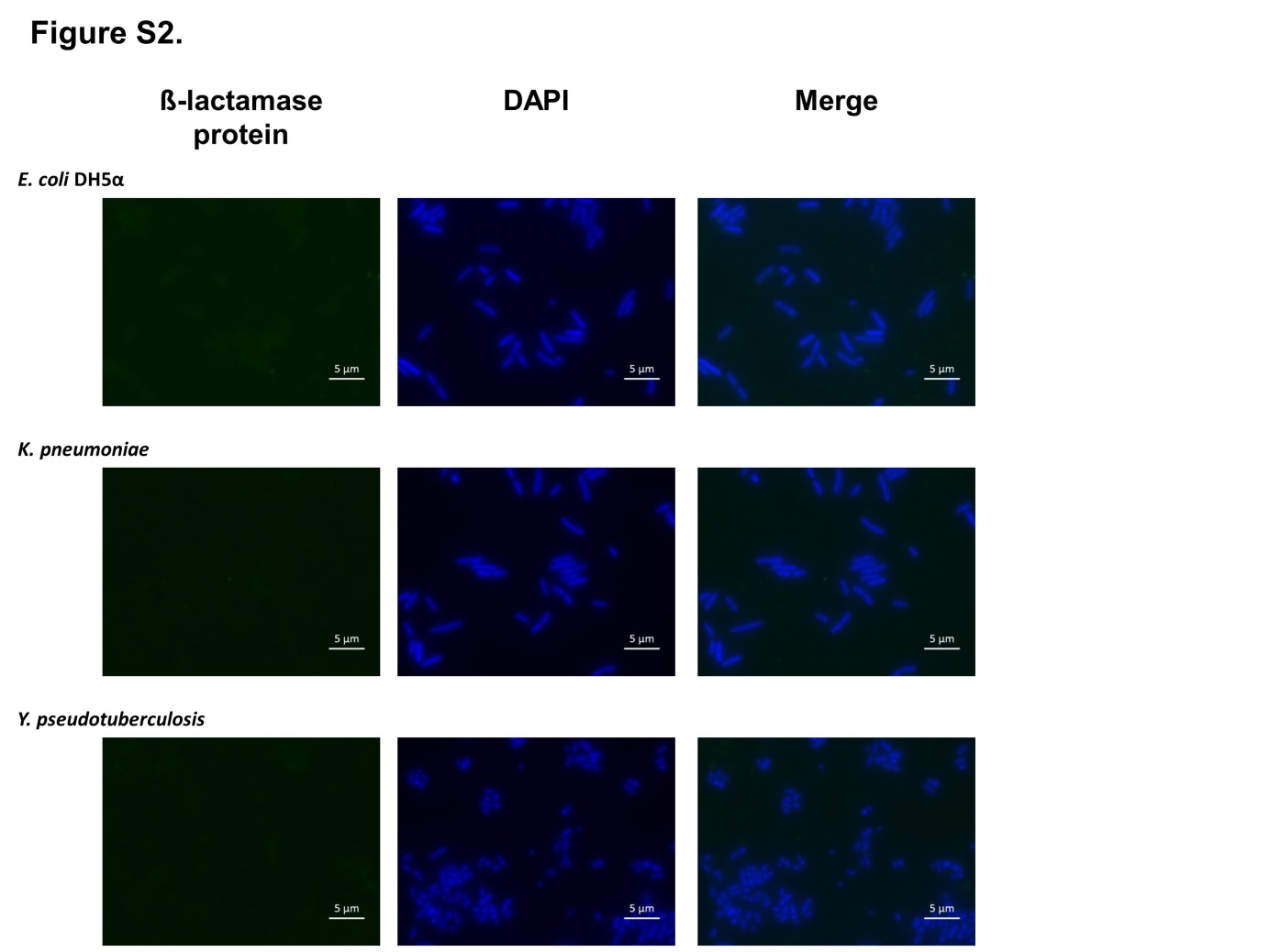


Antibody staining (green) of the TEM ß-lactamases in *E. coli* and DAPI (blue) staining. First row: *E. coli* DH5α. Second row: *K. pneumoniae*. Third row: *Y. pseudotuberculosis*.
