# Supplementary material for: Rapid screening for antibiotic resistance elements on the RNA transcript, protein and enzymatic activity level
Source: Ann Clin Microbiol Antimicrob. 2016 Sep 23;15:55. doi: 10.1186/s12941-016-0167-8 (PMC5035493; doi:10.1186/s12941-016-0167-8)
Supplement: Supplementary file 3 — 10.1186/s12941-016-0167-8 Examined environmental E. coli isolates with TEM-mediated ampicillin resistance. [file 12941_2016_167_MOESM3_ESM.docx]

**Supplementary Data III**

Additional File 3: Table S1: Examined environmental *E. coli* isolates with TEM-mediated ampicillin resistance

| Strain name | TEM-variant | Source | Country | FISH | Immunofluorescence |
| --- | --- | --- | --- | --- | --- |
| BFR-EC0702 | TEM-1 | Calf, feces | Germany | +/- | + |
| BFR-EC0715 | TEM-1 | Laying hen, feces | Germany | + | + |
| BFR-EC0716 | TEM-1 | Human | Germany | + | + |
| BFR-EC0766 | TEM-52 | Laying hen, feces | Germany | + | + |
| BFR-EC0805 | TEM-52 | Laying hen, feces | Germany | + | + |
| BFR-EC0843 | TEM-52 | Laying hen, feces | Germany | + | + |
| BFR-EC0883 | TEM-52 | Laying hen, feces | Germany | + | + |
| BFR-EC0900 | TEM-52 | Laying hen, feces | Germany | + | + |
| BFR-EC0975 | TEM-1 | Turkey hen, appendix | Germany | + | +/- |
| BFR-EC1347 | TEM-1 | Calf, feces | Germany | + | + |
| BFR-EC1379 | TEM-30 | Calf, feces | Germany | - | + |
| BFR-EC1417 | TEM-52 | Laying hen, feces | Germany | + | + |
| BFR-EC1504 | TEM-52 | Calf, feces | Germany | + | + |
| BFR-EC1510 | TEM-52 | Broiler, feces | Germany | + | + |
| BFR-EC1727 | TEM-52 | Broiler, feces | Germany | + | + |
| BFR-EC1864 | TEM-52 | Broiler, feces | Germany | + | + |
| BFR-EC2343 | TEM-52 | Broiler, feces | Germany | - | +/- |
| BFR-EC0084 | TEM-52 | Laying hen, feces | Germany | + | + |
| BFR-EC0239 | TEM-52 | Fattening pig, feces | Germany | +/- | + |
| BFR-EC0389 | TEM-1 | Broiler, feces | Germany | + | + |
| BFR-EC0561 | TEM-52 | Chicken | Germany | + | +/- |
| BFR-EC0564 | TEM-52 | Chicken | Germany | + | + |
| BFR-EC0577 | TEM-1 | Turkey hen, feces | Germany | +/- | +/- |
| BFR-EC0860 | TEM-52 | Unavailable | Germany | + | +/- |
| BFR-EC1240 | TEM-1 | Chicken | Germany | + | +/- |
| BFR-EC1828 | TEM-52 | Broiler, feces | Germany | + | +/- |

+: strong signals, +/-: weaker, but still sufficient signals, -: signal strength too low for reliable results
