# Supplementary material for: Rapid screening for antibiotic resistance elements on the RNA transcript, protein and enzymatic activity level
Source: Ann Clin Microbiol Antimicrob. 2016 Sep 23;15:55. doi: 10.1186/s12941-016-0167-8 (PMC5035493; doi:10.1186/s12941-016-0167-8)
Supplement: Supplementary file 4 — 10.1186/s12941-016-0167-8 Detection of ß-lactamase activity by the nitrocefin assay. [file 12941_2016_167_MOESM4_ESM.docx]

**Supplementary Data IV**

Additional File 4: Figure S3: Detection of ß-lactamase activity by the nitrocefin assay


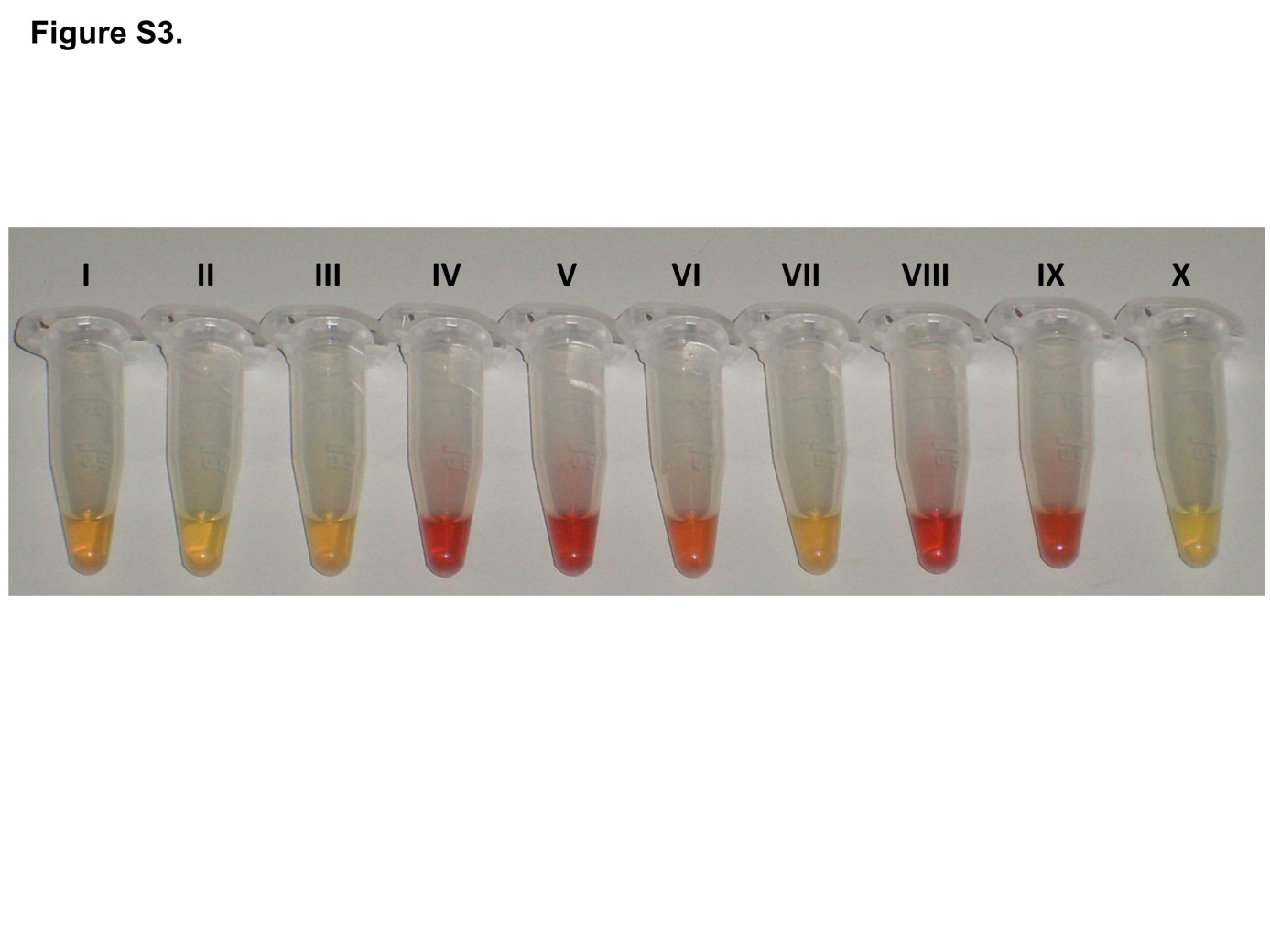


From the left to the right: *E. coli* GeneHogs (I), *E. coli* DH5α (II), *E. coli* pBR328 (III), *E. coli* pLitmus38 (IV), *E. coli* pUC18 (V), *E. coli* ATCC 35218 (VI), *K. pneumoniae* K2 (VII), *K. pneumoniae* My6107 (VIII), *Y. enterocolitica* (IX), *Y. pseudotuberculosis* (X). Red colour indicates resistance against ß-lactam antibiotics; pictures were taken 15 min after adding nitrocefin.
